# Frequency of Beta-Lactamase Antibiotic Resistance Genes in *Escherichia Coli* and *Klebsiella pneumoniae*

**DOI:** 10.4314/ejhs.v31i3.24

**Published:** 2021-05

**Authors:** Roghieh Golsha, Maryam Montazeri, Nazanin Razaghi, Mina Einollah Zade

**Affiliations:** 1 Infectious Diseases Research Center, Golestan University of Medical Science, Gorgan, Iran; 2 General Practitioner, Infectious Diseases Research Center, Golestan University of Medical Science, Gorgan, Iran; 3 Laboratory Sciences Research Centre, School of Medicine, Golestan university of Medical sciences, Gorgan, Iran; General Practitioner, Golestan University of Medical Sciences, Gorgan, Iran

**Keywords:** Antimicrobial Drug Resistance, Escherichia coli, Klebsiella, AmpC beta-lactamases

## Abstract

**Background:**

This cross-sectional study was performed on isolates of Klebsiella pneumoniae, and E.coli from clinical specimens of patients admitted to Sayyad Shirazi Hospital by census sampling method in 2019. Antibiogram testing was performed using the disk diffusion method as defined by the Clinical and Laboratory Standards Organization for performing this test. Finally, the abundance of genes was evaluated by PCR using specific primers. Frequency, percentage, mean±SD were used to describe the data. Chi-square and Fisher's exact tests were used to compare the presence and absence of the studied genes alone and in the presence of each other.

**Result:**

This study was performed on 130 positive samples, isolated from 32 (24.6%) males and 98 (65.4%) females with a mean age of 43.78 ± 21.72. From the total number of 130 isolates, 84 (64.6%) consisted of E.coli, and 46 (35.4%) were Klebsiella. Most of the cultures were urine and vaginal (61.5%). The highest antibiotic resistance in isolates was cephalexin and cefazolin (67.9% in E.coli & 63% in Klebsiella). Colistin was identified as the most effective antibiotic (100%) in both. AMPC extendedspectrum β-lactamase genes were present in 40 (30.8%) isolates. The highest frequency about the gene pattern of AMPC positive β-lactamase bacteria was correlated to DHA, FOX, and CIT genes, while none of the samples contained the MOX β-lactamase gene. E.coli and Klebsiella beta-lactamase-producing AMPC isolates were also significantly correlated with antibiotic resistance to the cephalosporin class (P <0.05).

**Conclusion:**

This study indicated a high percentage of resistance to third and fourth generation cephalosporins. Hence, careful antibiogram tests and prevention of antibiotic overuse in infections caused by AMPC-producing organisms and screening of clinical samples for the resistance mentioned above genes and providing effective strategies to help diagnose and apply appropriate treatments and change antibiotic usage strategies can partially prevent the transmission of this resistance.

## Introduction

Infectious diseases and their treatment have become one of the major issues of human society since the development of antimicrobialresistant strains (1, 2). Infections caused by resistant organisms are common and important causes of morbidity and mortality, disability, prolonged hospitalization, imposition, and increased health expenses and problems. These infections are difficult to treat, sometimes leading to patients' death, and are considered an increased risk. Gram-negative bacilli are important organisms causing nosocomial infections and are among the major causes of morbidity and mortality in patients (3, 4).

One of the main classes of bacteria causing these infections is the *Enterobacteriaceae* family. This family's two critical genera include *Escherichia coli* (E.*coli*) and *Klebsiella*, both opportunistic nosocomial pathogens (5, 6). These two genera, especially *E.coli* and *Klebsiella pneumoniae,* are opportunistic pathogens that cause septicemia, bacteremia, infantile enteritis, meningitis, and urinary tract infections of soft tissues. These human pathogens are responsible for infections and diseases in hospitalized patients with immunodeficiency and underlying conditions such as diabetes mellitus and chronic pulmonary disorders (7, 8).

β-lactams are among the most widely used antibiotics for treating bacterial infections that inhibit the trans-peptidase enzyme, disrupting cell wall synthesis and, ultimately, bacterial death (9). β-lactamase enzymes are produced by these bacteria and destroy the β-lactam ring in the β-lactam antibiotics (10). A group of β-lactamases is known as extended-spectrum β-lactamases. These enzymes have three phenotypes called extended-spectrum β-lactamases (ESBLs), Metalo-β-lactamases (MBLSs), and AMPC β-lactamases (11). AMPC β-lactamase enzymes are encoded by chromosomes or plasmids and affect a wide range of β-lactam drugs. The genes in the AMPC β-lactamases plasmids include either *MOX, CIT, DHA, ACC, EBC, FOX* genes, one of which is widely expressed in each geographical region (12).

Antibiotic resistance is a global health problem, but its propagation in developing countries is still unknown. Therefore, these enzymes' in vitro detection is essential in preventing the clinical failures caused by inappropriate antimicrobial treatment teams (13).

The presence of broad-spectrum betalactamases in organisms has caused many therapeutic problems and led to their resistance to potent antibiotics such as aminoglycosides and quinolones. This leads to longer hospitalization, increased mortality rate and treatment costs compared to antibiotic-sensitive microorganisms. Due to the lack of awareness about the prevalence of the infections caused by *E.coli* and *Klebsiella pneumoniae* producing AMPC β-lactamases plasmids in Gorgan city (Iran), and the pattern of spread of antibiotic resistance plasmids in the region we decided to conduct a study to investigate the antibiotic resistance genes of β-lactamases in *E. coli* and *Klebsiella pneumoniae* isolates from the patients in Shahid Sayyad-E-Shirazi Hospital of Gorgan (Iran).

## Materials and Methods

**Study design**: This cross-sectional study was performed on *Klebsiella pneumoniae,* and *Escherichia coli* isolates from clinical specimens of patients admitted to Shahid Sayyad-E-Shirazi Hospital using the census sampling method during 2019. Sampling from the hospitalized patients was performed by hospital staff according to the standards of the hospital.

**Laboratory procedures**: The specimens were cultured in the hospital's microbiology laboratory and assessed by the antibiogram test if positive. For isolates including Amoxicillin (25 µg and 10 µg), Ceftriaxone (30 µg), Ciprofloxacin (30 µg), Amikacin (30 µg), Nalidixic acid (30 µg), Cefalexin (30 µg), Norfloxacin (10 µg), Ampicillin (10 µg), Cefalotin (30 µg), Ampicillin (10 µg), Gentamicin (10 µg), Cefotaxime (30 µg), Ceftazidime (30 µg), Co-trimoxazole (25 µg), and Imipenem (10 µg), the antibiogram test was performed using Clinical and Laboratory Standards Institute (CLSI) standard (14). After transferring the samples to the laboratory for purification from secondary contaminations and then storing them, cultures were incubated on Blood Agar and MacConkey Agar for 24 hours at 37 ° C. If gram-negative coccobacillus was observed in gram staining and in case of observing oxidase-negative isolates in the oxidase test, these bacteria were cultured in EMB, SIM, TSI, urea and citrate medium for further biochemical tests.

In the next step, the combination disk test with phenylboronic acid was performed to identify the presence of AmpC in the isolates phenotypically. In this method, the microbial suspension was first cultured on the molar Hinton agar medium. Then ceftazidime, ceftazidime / phenylboronic acid, cefotaxime, and cefotaxime / phenylboronic acid discs were placed on the medium. The distance between the combination discs had to be 20 mm. The antibiotics concentration in the discs was 30 µg, and the phenylboronic acid concentration was 400 µg. Increasing the inhibition zone's diameter around each disc containing phenylboronic acid compared to the discs lacking it (equal to or greater than 5 mm) indicated the presence of AMPC-type β-lactamases. *E. coli ATCC2599* and *Staphylococcus aureusATCC 25923* were used as standard strains for the quality control of the antibiogram tests.

For the Polymerase Chain Reactions (PCRs), the bacterial DNA was extracted using the CinnaGen™ kit. PCR reactions were performed using the following specific primers to detect β-lactamase genes in AMPC plasmids (15). The program of the thermocycler's device for studying genes was as follows: The*E.coli ATCC2599* containing the mentioned genes was used as a positive control. PCR-specific water samples were also used as a negative control in all reactions.

**Statistical analysis**: The SPSS software version 18 was used for data analysis. The frequency, percentage, mean, and standard deviation were used for describing the data. *Chi*-square and Fisher's accurate tests were used to evaluate and compare the desired genes' presence and absence alone and together. The *P*-value of less than 0.05 is considered statistically significant.

## Results

This study was performed on 130 positive samples, isolated from 32 (24.6%) male and 98 (65.4%) female individuals (Table 1).

**Table 1:** Frequency of sex and distribution of AMPC-positive β-lactamase isolates in Patients with Positive Culture of *E.coli* and *Klebsiella* Organisms.

	Male	Female	Type of AMPC positive organism	
			
			<one Week	≥one week
*Escherichia coli*	17(20.2%)	67(79.8%)	15(68.2%)	7(31.8%)
*Klebsiella pneumoniae*	15(6.32%)	31(67.4%)	7(53.8%)	6(46.2%)

From the total number of 130 isolates, 84 (64.6%) consisted of *E.coli,* and 46 (35.4%) were *Klebsiella*. The mean age of patients in this study was 43.78 ± 21.72. The most frequent age groups from which *E.coli* and *Klebsiella* samples were isolated were the 25–40 years (32.1 % in *E.coli*& 32.6% in *Klebsiella*). Generally, in the two cultures of *E.coli* and *Klebsiella,* positive results were obtained from urine and vaginal cultures, and only one case of ascites and trachea was reported (Table 2).

**Table 2:** Frequency of source and hospital wards regarding positive samples of *E.coli* and *Klebsiella* Organisms

Sample	*Escherichia coli*	*Klebsiella pneumoniae*	Total
***Source of samples***			

Urine	31(36.9%)	14(30.4%)	45(34.6%)
Vaginal	22(26.2%)	13(28.3%)	35(26.9%)
Rectum	15(17.9%)	3(6.5%)	18(13.8%)
Wounds	8(9.5%)	3(6.5%)	11(8.5%)
Sputum	2(2.4%)	7(15.2%)	9(6.9%)
Blood	15(17.9%)	3(6.5%)	7(5.4%)
Eye	1(1.1%)	2(4.3%)	3(2.3%)
Ascites	1(1.1%)	0(0%)	1(0.8%)
Trachea	1(1.1%)	0(0%)	1(0.8%)
***Hospital wards***			
Maternity	38(45.2%)	16(34.8%)	54(41.5%)
Emergency	13(15.5%)	4(8.7%)	17(13.1%)
Lung	6(7.1%)	4(8.7%)	10(7.7%)
Nephrology	5(6%)	3(6.5%)	8(6.2%)
General	6(7.1%)	2(4.3%)	8(6.2%)
Infectious	5(6%)	2(4.3%)	7(5.4%)
ICU	0(0%)	6(13%)	6(4.6%)
NICU	1(1.2%)	3(6.5%)	4(3.1%)
Post CCU	2(2.4%)	0(0%)	2(1.5%)
Digestion	1(1.2%)	0(0%)	1(0.8%)
Neurology	0(0%)	1(2.2%)	1(0.8%)
Hematology	1(1.2%)	0(0%)	1(0.8%)

ICU: Intensive care unit, NICU: neonatal intensive care unit, CCU: coronary care unit

According to Table 2, most of the *E.coli* positive samples were collected from the maternity and emergency wards, and the majority of *Klebsiella* positive samples were obtained from the maternity ward, followed by the intensive care unit (ICU).

Based on the duration of hospitalization, patients were divided into two groups of less than one week and more than one-week hospitalization, and the result showed that the frequency of patients with less than one-week hospitalization was higher (63.8%).

According to the antibiogram test results and antibiotic resistance pattern of *E.coli* and *Klebsiella*, it was revealed that the highest percentage of antibiotic resistance in these bacteria was correlated to Cephalexin, Cefazoline, and carbenicillin. Colistin was identified as the most effective antibiotic (100%) in both microorganisms as well (Table 3).

**Table 3:** Frequency of Antibiogram of positive samples of *E.coli* and *Klebsiella* organisms.

Antibiogram	Resistant	
	
	*Escherichia coli*	*Klebsiella pneumoniae*
Carbenicillin	52(60.7%)	27(60%)
Piperacillin	50(59.5%)	29(63%)
Imipenem	11(13.4%)	8(17.8%)
Cefalexin	57(67.9%)	29(63%)
Cefazolin	57(67.9%)	29(63%)
Cefotaxime	42(50.6%)	24(52.2%)
Ceftazidime	39(47%)	23(50%)
Cefotiam	41(49.4%)	20(43.5%)
Cefixime	41(49.4%)	22(47.8%)
Ceftriaxone	39(47%)	25(54.3%)
Cefepime	25(30.1%)	15(32.6%)
Ciprofloxacin	38(43.6%)	23(50%)
Norfloxacin	37(44.6%)	21(45.7%)
Ofloxacin	37(44.6%)	21(45.7%)
Cotrimoxazole	39(47%)	26(56.6%)
Nalidixic Acid	46(55.4%)	24(52.2%)
Nitrofurantoin	38(43.6%)	19(41.3%)
Amikacin	22(26.5%)	16(34.8%)
Tobramycin	23(27.4%)	19(41.3%)
Gentamicin	23(27.4%)	15(32.6%)
Colistin	0 (0%)	0(0%)

From 130 isolates, 42 (32.3%) cases showed AMPC phenotype, 27 of 84 patients were *E.coli*positive, and 15 of 46 patients were *Klebsiella* positive isolates. The PCR reaction was performed to evaluate and confirm the presence of AMPC plasmid genes. The results showed that out of 130 isolates of these two bacteria mentioned above, AMPC extended-spectrum β-lactamase genes were present in 40 (30.8%) isolates and the rest of them lack these genes. The highest frequency about the gene pattern of AMPC positive β-lactamase bacteria was correlated to *DHA, FOX*, and *CIT* genes, while none of the samples contained the *MOX* β-lactamase gene.

In *E. coli,* only 25 isolates (29.8%) and *Klebsiella* 15 isolates (32.6%) contained AMPC plasmid. The results showed that *DHA* and *CIT* genes were positive in both *E.coli* and *Klebsiella* with similar percentages. The *MOX* gene was not present in any of the bacteria (Table 4). Besides, two isolates of *E.coli* contained two AMPC-coding β-lactamase genes (one of the isolates had *DHA* and *FOX* genes, and the other contained *CIT* and *FOX* genes), and one *Klebsiella* isolate carried the *ACC* and *DHA* genes simultaneously.

**Table 4:** Frequency of types of AMPC-positive beta-lactamase genes in *E.coli* and *Klebsiella* organisms

Type of organism	*Escherichia coli*	*Klebsiella pneumoniae*	Total
AMPC-positive plasmid	25(29.8%)	15(32.6%)	40(30.8%)
FOX - positive	11(13.1%)	2(4.3%)	13(10%)
EBC - positive	0(0%)	2(4.3%)	2(1.5%)
ACC - positive	2(2.4%)	2(4.3%)	4(3.1%)
DHA - positive	9(10.7%)	6(13%)	15(11.5%)
CIT - positive	5(6%)	4(8.7%)	9(6.9%)
MOX - positive	0(0%)	0(0%)	0(0%)

Table 5 represents the frequency of AMPC positive cases in both *E.coli* and *Klebsiella* based on the source of positive isolates culture. The frequency of AMPC positive in *E.coli* was from vaginal and urine samples, whereas in *Klebsiella*, positive culture extract was from sputum (Table 5). Most of the positive cases of the aforementioned extended-spectrum β-lactamases were found in the maternity ward (14 cases) following ICUs and emergencies.

**Table 5:** Frequency of AMPC positive beta-lactamase gene in *E.coli* and *Klebsiella* organisms by sample source

	Source	Organism	
		
		*Escherichia coli*	*Klebsiella pneumoniae*
AMPC positive	Blood	1(25%)	1(33.3%)
	Urine	11(35.5%)	3(21.4%)
	Sputum	0(0%)	4(57.1%)
	Wounds	2(25%)	0(0%)
	Vaginal	8(36.4%)	5(38.5%)
	Rectum	3(20%)	0(0%)
	Eye	0(0%)	2(100%)

The association between *E.coli* and *Klebsiella* containing AMPC β-lactamase and hospitalization duration was investigated using the *chi*-2 test, and a one-week hospitalization showed higher frequency (Table 1).

Investigating the relationship between AMPC β-lactamases and antibiotic resistance patterns showed that *E.coli* positive cultures containing the extended-spectrum β-lactamase showed significant antibiotic resistance to Piperacillin, Cephalexin, Cefazolin, Cefotaxime, Ceftazidime, Cefotiam, Cefixime, Ceftriaxone, Cefepime, Amikacin, and Tobramycin. Whereas in AMPC-producing *Klebsiella* bacteria, higher antibiotic resistance to Piperacillin, Cephalexin, Cefazolin, Cefotaxime, Ceftazidime, Cefotiam, Ciprofloxacin, Norfloxacin, Ofloxacin, and Cotrimoxazole was reported. (Table 6).

**Table 6:** Relationship between antibiotic resistance pattern in *E. coli* and *Klebsiella* pneumoniae with beta-lactamase containing AMPC

Antibiogram	*Escherichia coli*		*P.value*	*Klebsiella pneumoniae*		*P.value*
				
	*Ampc-Pos*	*Ampc-Neg*		*Ampc-Pos*	*Ampc-Neg*	
				
	*Resistant*			*Resistant*		
Carbenicillin	14(56%)	37(62.6%)	0.565	10(71.4%)	17(54.8%)	0.293
Piperacillin	20(80%)	30(50.8%)	0.013	13(86.7%)	16(51.6%)	0.211
Imipenem	3(12%)	8(14%)	1	4(26.7%)	4(13.3%)	0.41
Cefalexin	25(100%)	32(54.2%)	0.001	15(100%)	14(45.2%)	0.001
Cefazolin	25(100%)	32(54.2%)	0.001	15(100%)	14(45.2%)	0.001
Cefotaxime	22(88%)	20(34.5%)	0.001	12(80%)	12(38.7%)	0.009
Ceftazidime	22(88%)	17(29.4%)	0.001	11(73.3%)	12(38.7%)	0.028
Cefotiam	21(84%)	20(34.5%)	0.001	11(73.3%)	9(29%)	0.004
Cefixime	23(92%)	18(31%)	0.001	10(66.7%)	12(38.7%)	0.075
Ceftriaxone	21(84%)	18(31%)	0.001	11(73.3%)	14(45.2%)	0.075
Cefepime	12(48%)	13(22.4%)	0.021	6(40%)	9(29%)	0.514
Ciprofloxacin	14(56%)	24(40.7%)	0.197	12(80%)	11(35.5%)	0.005
Norfloxacin	14(56%)	23(39.7%)	0.169	10(66.7%)	11(35.5%)	0.047
Ofloxacin	14(56%)	23(39.7%)	0.169	10(66.7%)	11(35.5%)	0.047
Cotrimoxazole	14(56%)	25(43.1%)	0.281	12(80%)	14(45.2%)	0.025
Nalidixic Acid	17(68%)	29(50%)	0.131	6(40%)	15(50%)	0.526
Nitrofurantoin	15(60%)	23(40.4%)	0.1	8(53.3%)	11(35.5%)	0.249
Amikacin	14(56%)	12(20.3%)	0.018	6(40%)	10(32.3%)	0.605
Tobramycin	14(56%)	12(20.3%)	0.026	6(40%)	10(32.3%)	0.073
Gentamycin	9(36%)	13(22.4%)	0.249	8(53.3%)	8(25.8%)	0.191
Colistin	0(0%)	58(100%)	0(0%)	0(0%)	31(100%)	0(0%)

## Discussion

In this study, out of 130 bacterial isolates, positive cultures were mostly obtained from urine and vaginal samples collected from the maternity ward, emergency, and ICU units.

Generally, *E.coli*and *Klebsiella* are the most common cause of urinary tract infection in women (16). In 2014 Mansouri *et al.* reported that the prevalence of *E.coli* and *Klebsiella* in females with UTI was higher than males, probably due to the shortness of their urethra and its proximity to the vagina and anus (17). This study's results were consistent with the Al-Jebouri study conducted in Iraq and the study of Kaur *et al.* in Northern India. In these studies, the most common bacterial isolates were *E.coli* and were more prevalent in females (18,19).

Rahimi-Bashar*et al.* examined 10,332 patients and reported the highest frequency of *E.coli*, and *Klebsiella*'s positive cultures were isolated from Bloodstream (18%), Urinary tract (27%), and surgical site infection (17%), respectively (20). In Nwafia *et al.* study, out of the 200 *E. coli* isolates, 70 (35.00%) were confirmed positive for extended-spectrum betalactamase production, and urine samples (53.0%) had the highest frequency in *E.coli* cultures (21). These studies showed that the infection prevalence varies in different parts of hospitals in each hospital, which seems to be due to differences in hospitalization duration, antibiotic use, and health issues in the sections.

In the present study, the highest percentage of antibiotic resistance in *E.coli* and *Klebsiella* observed towards Cephalexin and Cefazolin antibiotics, and the Imipenem and Gentamicin were the lowest. In Ebrahim's study, AmpC β-lactamase-producing GNB showed high resistance rates to amoxicillin/clavulanate (92.1%), cefuroxime (91.1%), trimethoprim/sulfamethoxazole, and aztreonam (22). In a small-scale study, 33% of *E.coli* positive patients were resistant to cefotaxime (23). While the resistance to this antibiotic is close to 50% in the present study, this indicates an increase in its resistance in recent years.

Mansouri *et al.* assessed 338 isolates of *Escherichia coli* and 75 isolates of *Klebsiella* in Kerman. They reported that the highest antibiotic resistance was towards Ampicillin (91% in *E.coli*and 84% in *Klebsiella*), followed by Tetracycline and Nalidixic acid (24). The present study confirms the study conducted by Mansouri *et al.*. In this study, lower resistance of *Klebsiella* towards Gentamicin was reported. On the other hand, based on the different studies performed in Iran, it is found that the most effective antibiotic against *E.coli*and *Klebsiella* are generally Imipenem and Meropenem antibiotics (25, 26). Given the above results and despite the low antibiotic resistance toward these antibiotics, it should be noted that indiscriminate consumption of this antibiotic in treatment should be avoided in order to stop resistance increment.

During molecular studies performed by PCR analysis, the most prevalent were related to the *DHA* and *FOX* β-lactamase genes. At the same time, none of the isolates contained the MOX β-lactamase gene. However, in the Zorgani study in Tripoli, the majority of AmpC positive isolates (66.6%) were found to carry the CMY encoding gene, followed by MOX, DHA, and EBC(27).

Ribeiro *et al.* (28) and Kazemian *et al.* (29) identified DHA, CMY, and CIT pAmpC in *klebsiellapneumoniae, E. coli, and Proteus mirabilis* in different types of nosocomial infections in Portugal and Iran, respectively. The predominant gene was similar to the present study, and the overall prevalence of β-lactamase genes was higher in both studies (30). In a survey by Mohamudha *et al.*, the most prevalent genotype in both types (*Klebsiella and E.coli )* of isolates was CIT-DHA (31). In another report, DHA was more common in both *Klebsiellaspp.* and *E. coli* isolates (46.7% and 38%, respectively, no FOX was detected (32). In another study, out of 148 isolates, 33.8% of the isolates contained AMPC β-lactamase, 46% of which were *Klebsiella* isolates, and 22% were *E.coli*. The most common AMPC coding gene, *CMY-1*, was observed at 73.9%, and the *FOX* gene was observed in 40.9% and 32.3% of *Klebsiella* and *E.coli*isolates, respectively. However, the *ACC* gene was not found in any of the bacteria. In the present study, two isolates of *E.coli*and two isolates of *Klebsiella* expressed the *ACC* gene, which was less prevalent in our study (33). These reports have similarities and differences with our study, which is related to the infection control system and long-term hospitalization and bacterial isolates' geographical disparities.

In the present study, the most prevalent AMPC-containing β-lactamases cases included both bacteria collected from maternity wards, followed by *E.coli* isolates from emergency wards, and *Klebsiella* isolates from ICU. Zorgani *et al.* (27) reported that all AmpC positive strains were recovered from patients hospitalized in different intensive care units (ICUs). In comparison, Saffar *et al.*(34) showed that 19.23% of the isolates from ICUs carried plasmid-mediated AmpC genes. However, most specimens were isolated from the emergency departments (44.6%), mainly due to referral cases admitted in the hospital. Considering that in most cases, β-lactamase resistance genes are transmitted by genetic elements such as plasmids, so they are simply transported from one strain to another and can lead to the propagation of resistance in the hospital and another therapeutic environment. Since these plasmids have moderate prevalence in the common hospital isolates, paying particular attention to selecting the correct medications and the appropriate treatment duration and effective disinfection can prevent the spread of these plasmids among different bacteria.

*E.coli* and *Klebsiella* isolate containing AMPC were found to be significantly resistant to Cephalosporins (Cephalexin, Cefazolin, Cefotaxime, Ceftazidime, and Cefotiam). In a study performed in China, by investigating the antibiotic pattern of the AMPC-producing *Klebsiella* isolates, it was found that this bacterium is resistant to third- and fourth-generation Cephalosporins such as Ceftriaxone, Ceftazidime, and Cefepime. Moreover, it was highly resistant to Quinolones, Aminoglycosides, including Ciprofloxacin, Levofloxacin, and Amikacin. However, no such association was observed in Carbapenems (23). The results of this study are in line with the present study. It seems that third-generation Cephalosporins can rapidly cross the outer membrane and are generally used to treat infections caused by AMPC-producing bacteria. While in the Chinese study, high resistance to this category of medication is becoming a growing concern (35), the use of fourthgeneration Cephalosporins should, therefore, be used with caution based on the preliminary laboratory results. However, Liu Xo *et al.* (36). indicated that carbapenems' sensitivity and resistance were not significantly correlated with the AMPC gene's presence. It seems to be due to the presence of penicillin-binding proteins, and this antibiotic can treat the infections caused by AMPC-producing *Klebsiella*. Besides, in the present study, two AMPC-coding β-lactamase genes were found in three cases. Two *Escherichia coli* isolates contained *DHA, CIT,* and *FOX* genes, and one *Klebsiella* isolate had two AMPC-producing β-lactamase genes, including *FOX* and *ACC*. The presence of two genes at the same time may raise the concern that the transmissibility of resistance genes can occur more often in a hospital environment which could lead to the severity and deterioration of infections in patients. The emergence of β-lactamase enzymes among gram-negative bacteria, especially those that play an important role in nosocomial infections, is an important concern. The outbreak of multiple resistant strains has raised serious problems in terms of treatment and diagnosis of such strains.

This study was included with some limitations such as small sample size, which would lead to type II error. Hence, more comprehensive studies on larger sample size, including more antibiotics, is recommended.

In conclusion, according to the high percentage of resistance towards third and fourth generation cephalosporins, careful antibiogram tests and prevention of antibiotic overuse in infections caused by AMPC-producing organisms and screening of clinical samples for the resistance mentioned above genes and providing effective strategies to help clinical diagnosis and application of appropriate treatments and change antibiotic usage strategies can partially prevent the transmission of this resistance.
